# Using real-time polymerase chain reaction as an alternative rapid method for enumeration of colony count in live *Brucella* vaccines

**DOI:** 10.14202/vetworld.2017.610-615

**Published:** 2017-06-08

**Authors:** Waleed S. Shell, Mahmoud L. Sayed, A. A. Samy, Ghada Mohamed Al-Sadek, Gina Mohamed Mohamed Abd El-Hamid, Abdel Hakam M. Ali

**Affiliations:** 1 Central Laboratory for Evaluation of Veterinary Biologics, Cairo, Abbasia, Egypt; 2 Department of Microbiology and Immunology, National Research Center, Egypt

**Keywords:** *Brucella*, colony count, RB51, Rev-1, real-time polymerase chain reaction, S19, vaccines

## Abstract

**Aim::**

Brucellosis is a major bacterial zoonosis of global importance affecting a range of animal species and man worldwide. It has economic, public health, and bio-risk importance. Control and prevention of animal brucellosis mainly depend on accurate diagnostic tools and implementation of effective and safe animal vaccination program. There are three types of animal *Brucella* live vaccines - *Brucella melitensis* Rev-1 vaccine, *Brucella abortus* S19, and *B*. *abortus* RB51. Evaluation of these vaccines depends mainly on enumeration of *Brucella* viable count. At present, used colony count method is time consuming, costly and requires especial skills. Hence, the aim of this study is to use and standardize real-time polymerase chain reaction (RT-PCR) as an alternative, quantitative, sensitive, and rapid method to detect the colony count of *Brucella* in live *Brucella* vaccine.

**Materials and Methods::**

Four batches of different live *Brucella* vaccines were evaluated using of conventional bacterial count and RT-quantitative PCR (RT-qPCR) using BSCP31 gene specific primers and probe. Standard curve was generated from DNA template extracted from 10-fold serial dilution of living *B. abortus* RB51 vaccine to evaluate the sensitivity of RT-qPCR.

**Results::**

Results revealed that three batches of living *Brucella* vaccines were acceptable for *Brucella* colony count when traditional bacterial enumeration method was used. Results of RT-qPCR were identical to that of conventional bacterial count.

**Conclusions::**

Results concluded that RT-qPCR was relatively sensitive compared to traditional bacterial colony count of these vaccines.

## Introduction

Brucellosis is a major bacterial zoonosis of global importance affecting a range of different mammals including cattle, sheep, goats, swine, rodents, marine mammals, and man worldwide. In food animals, the disease primarily affects the reproductive system with concomitant loss in fertility and productivity of affected animals. In man, infection is characterized by recurrent febrile episodes that lead to the description of this disease as undulant fever (economic and public health importance) [1]. The severity of this disease and lack of vaccines suitable for use in man has led to the investigation of *Brucella* as agents for bioterrorism (bio-risk importance) [2]. Vaccines to be used for human are not yet available, and so eradication of human brucellosis largely depend on the eradication of the disease in animals. Eradication of brucellosis in animals has been a goal for many countries. To control brucellosis, comprehensive vaccination, surveillance, and quarantine programs should be implemented. Both control and prevention procedures are highly dependent on accurate diagnostic tools and implementation of effective and safe animal vaccination programs [3].

There are three types of animal *Brucella* live vaccines - *Brucella melitensis* Rev-1 vaccine (0.5-2×10^9^ colony forming unit [CFU]/dose) for vaccination of sheep and goats, *Brucella abortus* S19 (0.5-5×10^9^ CFU/dose) for vaccination of cattle and buffaloes, and *B. abortus* RB51 (1-3.4×10^10^ CFU/dose) for vaccination of cattle and buffaloes. Evaluation of these vaccines depends mainly on enumeration of viable count, smoothness or roughness, safety test and potency test [4]. European Pharmacopoeia [5] reviewed that the dose of Rev-1 vaccine in sheep and goats should contain not fewer than 0.5×10^9^ and not more than 4×10^9^ live bacteria per dose.

At present, practiced colony count method is time consuming, costly and requires especial skills. Hence, the aim of this study was to use and standardize real-time polymerase chain reaction (RT-PCR) as a quantitative, sensitive, and rapid method to detect the colony count of live *Brucella* vaccine.

## Materials and Methods

### Vaccines

Eight lyophilized living *Brucella* vaccines of different batches (two *B. abortus* S19, four *B. abortus* RB51, and two *B. melitensis* Rev-1). The lyophilized vaccines were reconstituted in vaccine diluents and were used for bacteriological colony count and genomic DNA extraction.

### Bacterial colony count of living *Brucella* vaccines

About 0.1 ml of expected countable dilutions of different live *Brucella* vaccines were inoculated in five plates of tryptone soya agar and spread with a sterile glass. CFU per vaccine dose were enumerated according to protocols described previously [4,6].

### Extraction of genomic DNA from *Brucella* strains

Genomic DNA extraction from single dose of live *Brucella* vaccines for evaluation of *Brucella* viable count and from 10-fold serial dilutions of RB51 vaccine from 10×10^10^ to 10×10^8^ CFU/ml for generation of standard curve (RB51 vaccine vial of 5 doses 2×10^10^/dose were reconstituted on 1 ml as 10×10^10^ CFU/ml and the other one reconstituted on 1 ml for serial dilution). Genomic DNA extraction was performed using G-spin Total DNA Extraction Kit (*iNtRON)* following the kit manufacturer’s protocol.

### Oligonucleotide primers and probes used in RT-PCR

**Figure-1:**
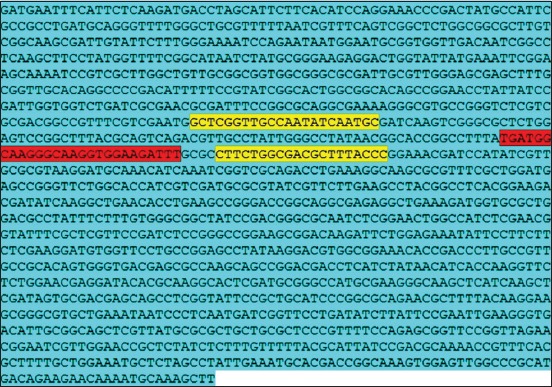
Genbank: BCSP31 KDa gene sequence (GenBank accession number M20404), https://www.ncbi.nlm.nih.gov/nuccore/M20404, showing forward and reverse primers (yellow color) and probe (red color).

Real-time PCR on tested samples was done using the primers and probe [7,8] identifying and targeting the bcsp31 gene (GenBank accession number M20404) [7] (Table-1 and Figure-1).

**Table-1:** RT-PCR oligonucleotides primers and probe of BCSF31 for *Brucella* species.

Primer	Sequence (5’ - 3’)	Amplicon size (bp)
BSCP31 Forward primer	GCTCGGTTGCCAATATCAATGC	151 bp
BSCP31 Reverse primer	GGGTAAAGCGTCGCCAGAAG	
RT-PCR probe	AAATCTTCCACCTTGCCCTTGCCATCA-FAM/BHQ1	

RT-PCR=Real-time polymerase chain reaction

### RT-PCR

RT-PCR assay was standardized and performed in Stratagene MX3005P quantitative PCR (qPCR) system. The PCR Master Mix and PCR cycling conditions used are given in Tables-2 and 3.

**Table-2:** Preparation of PCR master mix.

Component	Volume/reaction
2x QuantiTect Probe RT-PCR master mix	12.5 μl
Forward primer	0.2 μl (200 mm)
Reverse primer	0.2 μl (200 mm)
Probe	0.1 μl (100 μm)
DNase free water	6.8 μl
Template DNA	5 μl

RT-PCR=Real-time polymerase chain reaction

**Table-3:** RT-PCR cycling conditions.

Stage	Temperature	Time	Cycles
Primary denaturation	95°C	10 min	1
Amplification			
Secondary denaturation	95°C	30 s	40
Annealing and extension	60°C	90 s (optics on)	

RT-PCR=Real timepolymerase chain reaction

### RT-PCR standard curves

Standard curves were generated by plotting the cycle threshold values (CT) of the RT-qPCR performed on 10-fold serial dilutions of purified DNA from 10-fold serial dilutions of *B. abortus* RB51 vaccine (10×10^10^-10×10^8^ CFU/ml) against the log input cells/ml [9]. *Brucella* species concentrations were determined by the viable cell plate count method as mentioned above [4,6].

## Results and Discussion

In the absence of effective and safe human vaccine against brucellosis, animal vaccination against brucellosis is an important issue in control and eradication of brucellosis in animals and human. For more than 60 years, *B. abortus* S19 vaccine for buffaloes and cattle and *B. melitensis* Rev-1 vaccine for goats and sheep remain as the most efficient *Brucella* vaccines, and their use is of a great impact on the control and incidence of brucellosis in domestic ruminants and humans [10]. S19 and Rev-1 vaccines are used in vaccination of calves and ewes, respectively, in a dose of 0.5-2×10^9^ CFU/dose and 0.5-5×10^9^ CFU/dose [4]. Rev-1 vaccine can be used in a dose of 0.5-5×10^9^ CFU/dose [5]. RB51 vaccine strain was developed in 1982 by Prof. Gerhardt Schurig’s group and is derived from a virulent smooth *B. abortus* biovar 1 strain 2308. RB51 vaccine is used in vaccination of cows in a dose of 1-3.4×10^10^ CFU/dose [4,11]. Evaluation of these vaccines depends mainly on identification of vaccinal strains, enumeration of *Brucella* viable count, safety and potency. Enumeration of *Brucella* viable count is time consuming and needs special skills [4]. This study was designed to use a RT-qPCR as alternative, sensitive, and rapid method to detect colony count in *Brucella* vaccines. Eight batches of *Brucella* vaccines, two *B. abortus* S19, four *B. abortus* RB51, and two *B. melitensis* Rev-1 were evaluated by conventional bacterial colony count and RT-qPCR.

By using conventional colony count, seven batches of living *Brucella* vaccines used in this study were with satisfactory results and within the standard international range of acceptable dose for animal’s vaccination. On the other hand, one of the RB51 vaccine batches was unacceptable with colony count of 6×10^9^ CFU/dose as shown in Table-4.

**Table-4:** *Brucella* count by traditional methods and RT-qPCR based on a standard graph generated by *brucella* RB51 DNA within the range.

*Brucella* vaccines samples	CT	Estimation of *brucella* vaccines by rt-PCR	*Brucella* viable count by traditional methods	Acceptance
RB51	21.69	2.89×10^10^ CFU/dose	3.4×10^10^ CFU/dose	Accepted
RB51	23.00	1.25×10^10^ CFU/dose	3.4×10^10^ CFU/dose	Accepted
RB51	22.14	2.325×10^10^ CFU/dose	1.2×10^10^ CFU/dose	Accepted
RB51	28.65	4.19×10^9^CFU/dose	6×10^9^ CFU/dose	Not accepted
S19	27.98	5.025×10^9^ CFU/dose	4×10^9^ CFU/dose	Accepted
S19	31.52	1.6×10^9^ CFU/dose	4.8×10^9^ CFU/dose	Accepted
Rev-1	27.87	5.163×10^9^ CFU/dose	3×10^9^ CFU/dose	Accepted
Rev-1	32.00	1×10^9^ CFU/dose	1.5×10^9^ CFU/dose	Accepted

RT-qPCR=Real-time quantitative polymerase chain reaction, CFU=Colony forming unit, CT=Cycle threshold

In this study, bcsp31 gene was selected to be used in RT-qPCR for evaluation of colony count of living *Brucella* vaccines which is highly conserved gene among *Brucella* species and also used frequently as a gene target for diagnosis of human brucellosis [12-14], and therefore could potentially detect *B. melitensis* and *B. abortus* strains which were included in this study [15,16]. Moreover, it is specific method as it did not amplify DNA from any non-*Brucella* templates. The bcsp31 PCR was found to be 100% specific and was the most sensitive assay when compared with to omp2 and the 16S rRNA PCR [17]. BSCP31 PCR was used by many researchers for specific identification of genus *Brucella* from seropositive, active, relapsing, chronic cases in humans [18-20]. Furthermore, this gene target has been used specifically to detect *Brucella* in human cerebrospinal fluid, blood, and serum [21-23], in clinical tissues from seals [24] and in buffalo milk [25]. Many reports have been published on the diagnostic efficiency of qPCR assays using bcsp31 gene for diagnosis of brucellosis in human samples [26] and also used for screening of brucellosis from camel serum [27].

**Figure-2:**
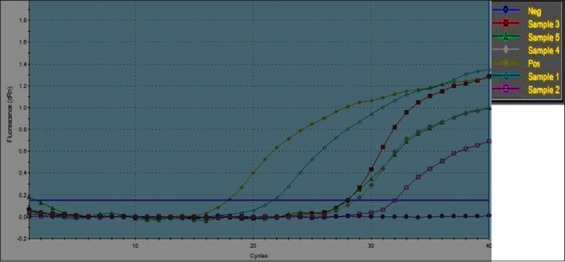
Amplification curves of real time-quantitative polymerase chain reaction for quantification of *Brucella* vaccine batches. Sample 1=RB51 vaccine, sample 2=Rev-1 vaccine, sample 3=RB51 vaccine, sample 4=S19 vaccine and sample 5=S19 vaccine.

**Figure-3:**
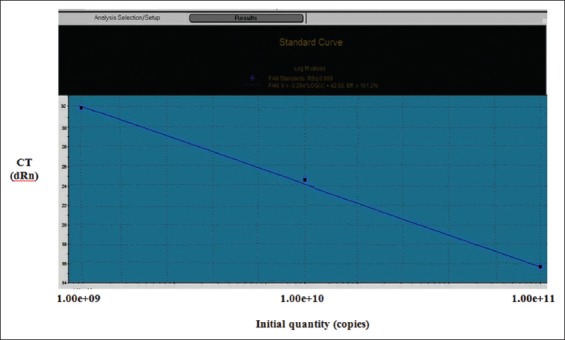
Schematic standard curve of a dilution series, plotting cycle threshold values over log template concentrations. The slope is used to estimate number of *Brucella* colonies/vaccine samples.

TaqMan technology determines the PCR cycle at which the increase in fluorescence of the reporter dye reaches a CT is proportional to the log of the amount of target DNA and hence the log of the number of bacteria in the sample. Standard graph was based on *B. abortus* RB51 DNA extracted from tenfold serial dilution of RB51 vaccine (Figure-2). The RT-PCR assay with primers and probe specific for the *Brucella* BCSP31 gene was positive for all vaccine samples. The CT values were clearly inversely related to the quantity of organisms, especially during standard curve generation. These CT values corresponded to 10^11^ CFU (positive at CT=16) to 10^9^ CFU (positive at CT=32) of *Brucella* organism when the values were fit into the standard curve generated by using the results for serial dilutions of RB51 vaccine. Colonies count 10^11^–10^9^ CFU represents the range of acceptable colonies count of all type of *Brucella* vaccines (1×10^9^ CFU/dose in case of Rev-1 vaccine to 3.4×10^10^ CFU/dose in case of RB51 vaccine) (Figure-3).

As shown in Figures-2 and 3 and Table-4, results of RT-qPCR were in agreement with results of traditional bacterial colony count except with one batch of Rev-1 vaccine where results of traditional colony count and RT-qPCR were 3×10^9^/CFU/dose and 5.163×10^9^/CFU/dose, respectively, but still results within the same log. Results of qPCR were with sensitivity of 87.5%. Findings confirmed that the unaccepted batch of RB51 vaccine by traditional colony count was out of standard international range of *B*. *abortus* RB51 vaccine.

Results agree with Angel *et al*. [28] who used RT-qPCR for enumeration of acetic acid bacteria with 100% sensitivity when compared with plating and microscope counting also was in agreement with Chaloemnon *et al*. [29] who enumerated the gastrointestinal microbiota (*Lactobacilli*, *Bifidobacteria*, and *Escherichia coli*) in weaning pigs by conventional culture and RT-PCR. Aline *et al*. [30] and Susan *et al*. [9]. Found high agreement with the results of traditional colony count and RT-qPCR when used to enumerate *Lactobacillus helveticus* in dairy products and *Streptococcus pneumoniae*, respectively.

In disagreement with these results, Botaro *et al*. [31] reviewed that the qPCR protocol can be used as a rapid diagnostic assay to accurately detect *Staphylococcus aureus* from bovine milk, but this protocol is not accurate for counting of *S*. *aureus* in bronopol-preserved milk samples from naturally infected mammary glands. Same findings were revealed from enumeration of living *E. coli* O157:H7 on plants [32].

A major drawback of qPCR is its inability to differentiate the DNA from viable and dead cells, and this is a critical factor for many researches’ especially in the food industry, water pollution researches, so to remedy this shortcoming, researchers have used biological dyes such as ethidium monoazide and propidium monoazide to pre-treat samples before DNA extraction which is important issue especially in food industry [32,33].

Results of this study may be more applicable than other studies which used RT-PCR for identification of organisms from tissues, water, etc., which may give false results due to nonspecific reactions which especially occurs when RT-PCR used for identification of multiple organisms using universal primers sets. However, in this study, we evaluated vaccines which contain one organism type (*Brucella*) as these vaccines were tested for sterility before counting process. Furthermore, although RT-PCR measure the total number of living and dead (dead cells as a results of freeze drying process) *Brucella* cells, but due to all these vaccines are subjected to the same factors as freeze drying program so ratio of living to dead cells were nearly constant and so it would not have an effect on the sensitivity of the RT-PCR.

## Conclusions

In this study, RT-qPCR assay was developed to enumerate colony count in live *Brucella* vaccines using DNA template extracted from tenfold serial dilutions of different living *Brucella* vaccines. The assay proved to be highly specific and sensitive when compared with traditional bacterial colony count of these vaccines. However, it needs more standardization, validation, and evaluation by using more batches of different live bacterial vaccines such as *Brucella* vaccines, *E. coli* (Poulvacpoultry vaccine), *Salmonella* vaccine (Megan VAC-1, poultry vaccine), and *Streptococcus equi* vaccine (PINNACLE^®^ I.N, horse vaccine) and evaluate the sensitivity of standard curves generated from DNA template extracted from tenfold serial dilutions of different living bacterial vaccines and from 10-fold serial dilution of template DNA.

## Authors’ Contributions

All authors designed and planned this research work. DNA extraction from different live *Brucella* vaccine batches and from serial dilution of control positive live *Brucella* vaccine batch were carried out by AAS, GMA, GMMAE and AMA. Traditional Colony count of different live *Brucella* vaccines batches was performed by all authors. RT-PCR on different live *Brucella* vaccines batches and construction of standard curve to estimate bacterial count in vaccine batches were carried out by WSS, MLS, AAS and AMA. All authors contributed equally in preparation and revision of the manuscript and collection of scientific papers related to the subject of this research. All authors read and approved the final manuscript.

## References

[1] Vipan K, Parveen K, Heigo P, Hanish S, Wadhawan V.M (2015). Brucellosis:A neglected enemy. Int. J. Curr. Med. Pharm. Res.

[2] Noormohamad M, Mohammad R.P (2016). Vaccines and vaccine candidates against brucellosis. Infect. Epidemiol. Med.

[3] Corbel M.J (2006). 2006 in humans and animals. World Health Organization Collaboration with the Food and Agriculture Organization of the United Nations and World Organization for Animal Health.

[4] OIE (2016). 2016 (*Brucella abortus**Brucella melitensis* and *Brucella suis*). Manual of Diagnostic Tests and Vaccines for Terrestrial Animals.

[5] European Pharmacopoeia (2005). 2005 Notices (1. 1 to All Monographs and other Texts, Brucellosis Vaccine (Live) (*Brucella melitensis* Rev. 1 Strain), Freeze-Dried, for Veterinary Use. European Pharmacopoeia, 04/2005:0793.

[6] Alton G.G, Jones L.M, Angus R.D, Verger J.M (1988). 1988 for the Brucellosis Laboratory.

[7] Elena A, Mohammad F, Tamer E, Israr S, Kamel A, Issa R, Assad M, Stelian B, Maria R.G, Doina D (2016). Validation of RT-qPCR technique for detection of *Brucella* genome in milk sheep and goat in west bank part of palestine. Sci. Bull. Ser. F Biotechnol.

[8] Probert W, Schrader K, Khuong N, Bystrom S, Graves M (2004). Real-time multiplex PCR assay for detection of *Brucella* spp *B. abortus B. melitensis*. J. Clin. Microbiol.

[9] Susan C.M, Jim F.H, David R.M, Anthony G.S.J (2014). Making standards for quantitative real-time pneumococcal PCR. Biomol. Detect. Quantif.

[10] Blasco J.M (1997). A review of the use of *B. melitensis* Rev 1 vaccine in adult sheep and goats. Prev. Vet. Med.

[11] Elaine M.S.D, Nammalwar S, Andrey P.L (2015). Recent advances in *Brucella abortus* vaccines. Vet. Res.

[12] Al Dahouk S, Fleche P.L, Nockler K, Jacques I, Grayon M, Scholz H.C, Tomaso H, Vergnaud G, Neubauer H (2007). Evaluation of *Brucella* MLVA typing for human brucellosis. J. Microbiol. Methods.

[13] Navarro E, Escribano J, Fernandez J.A, Solera J (2002). Comparison of three different PCR methods for detection of *Brucella* spp. In human blood samples. FEMS Immunol. Med. Microbiol.

[14] Morata P, Queipo-Ortuno M.I, Reguera J.M, Garcia-Ordonez M.A, Cardenas A, Colmenero J.D (2003). Development and evaluation of a PCR-enzyme linked immunosorbent assay for diagnosis of human brucellosis. J. Clin. Microbiol.

[15] Bricker B.J, Tabatabai L.B, Deyoe B.L, Mayfield J.E (1988). Conservation of antigenicity in a 31kDa *Brucella* protein. Vet. Microbiol.

[16] OIE (World organization of Animal Health) (2014). 2014 of Diagnostic Tests and Vaccines for Terrestrial Animals (Mammals, Birds and Bees).

[17] Mukherjee F, Jain J, Patel V, Nair M (2007). Multiple genus specific markers in PCR assays improve the specificity and sensitivity of diagnosis of brucellosis in field animals. J. Med. Microbiol.

[18] Kattar M.M, Zalloua P.A, Araj G.F, Samatha-Kfoury J, Shbaklo H, Kanj S.S, Khalife S, Deeb M (2007). Development and evaluation of real time polymerase chain reaction assays on whole blood and paraffin-embedded tissues for rapid diagnosis of human brucellosis. Diagn. Microbiol. Infect. Dis.

[19] Mitka S, Anetaki S.C, Souliou E, Diza E, Kansouzidou A (2007). Evaluation of different PCR assay for the early detection of acute and relapsing human brucellosis in comparison with conventional methods. J. Clin. Microbiol.

[20] Queipo-Ortuno M.I, Colmenero J.D, Bravo M.J, Garcia-Ordonez M.A, Morata P (2008). Usefulness of a quantitative real-time PCR assay using serum samples to discriminate between inactive, serologically positive and active human brucellosis. Clin. Microbiol. Infect.

[21] Debeaumont C, Falconnet P.A, Maurin M (2005). Real-time PCR for detection of *Brucella* spp. DNA in human serum samples. Eur. J. Clin. Microbiol. Infect. Dis.

[22] Colmenero J.D, Clavijo E, Morata P, Bravo M.J, Queipo-Ortuno M.I (2011). Quantitative real-time polymerase chain reaction improves conventional microbiological diagnosis in an outbreak of brucellosis due to ingestion of unpasteurized goat cheese. Diagn. Microbiol. Infect. Dis.

[23] Sohrabi M, Mobarez A.M, Behmanesh M, Khoramabadi N, Doust R.H (2011). Evaluation of a new set of real-time PCR for *Brucella* detection within human and animal samples. J. Pharm. Health Sci.

[24] Sidor I.F, Dunn J.L, Tsongalis G.J, Carlson J, Frasca S (2013). A multiplex real-time polymerase chain reaction assay with two internal controls for the detection of *Brucella* species in tissues, blood and feaces from marine mammals. J. Vet. Diagn. Invest.

[25] Amoroso M.G, Salzano C, Cioffi B, Napoletano M, Garofalo F, Guarino A, Fusco G (2011). Validation of a real time PCR assay for fast and sensitive quantification of *Brucella* spp. In water buffalo milk. Food Control.

[26] Sanjuan-Jimenez R, Morata P, Bermudez P, Bravo M.J, Colmenero J.D (2013). Comparitive clinical study of different multiplex real-time PCR strategies for the simultaneous differential diagnosis between extrapulmonary tuberculosis and focal complications of Brucellosis. PLoS Negl. Trop. Dis.

[27] EI Behiry A (2014). Evaluation of diagnostic techniques and antimicrobial resistance of *Brucella* spp. Isolated from blood serum of camels and camel ranchers. J. Microbiol. Res.

[28] Angel G, Nuria H, Montserrat P, Albert M, Jose M.G (2006). Enumerationand detection of acetic acid bacteria by real-time PCR and nested PCR. FEMS Microbiol. Lett.

[29] Chaloemnon P, Chomnawang M.T, Junlapho W, Paraksa N (2016). Application of real-time PCR for quantifying gastrointestinal microbiota in weaning pigs influenced by dietary feed additive supplementation. Thai J. Vet. Med.

[30] Aline M, Helene B, Elisabeth E, Leo M, Irmler S (2017). Detection and enumeration of *Lactobacillus helveticus* in dairy products. Int. Dairy J.

[31] Botaro B.G, Cortinhas C.S, Março L.V, Moreno J.F.G, Silva L.F.P, Benites N.R, Santos M.V (2013). Detection and enumeration of *Staphylococcus aureus* from bovine milk samples by real-time polymerase chain reaction. J. Dairy Sci.

[32] Zeng D, Chen Z, Jiang Y, Xue F, Li B (2016). Advances and challenges in viability detection of foodborne pathogens. Front. Microbiol.

[33] Ju W, Moyne A, Marco M.L (2016). RNA-based detection does not accurately enumerate living *Escherichia coli* O157:H7 cells on plants. Front. Microbiol.

